# Migrasomal autophagosomes relieve endoplasmic reticulum stress in glioblastoma cells

**DOI:** 10.1186/s12915-024-01829-w

**Published:** 2024-01-30

**Authors:** Seon Yong Lee, Sang-Hun Choi, Yoonji Kim, Hee-Sung Ahn, Young-Gyu Ko, Kyunggon Kim, Sung Wook Chi, Hyunggee Kim

**Affiliations:** 1 Department of Biotechnology, Korea University, Seoul, Republic of Korea; 2 Institute of Animal Molecular Biotechnology, Korea University, Seoul, Republic of Korea; 3 Convergence Medicine Research Center, Asan Institute for Life Sciences, Asan Medical Center, Seoul, Republic of Korea; 4 Department of Life Sciences, Korea University, Seoul, Republic of Korea; 5 Department of Convergence Medicine, Asan Medical Center, University of Ulsan College of Medicine, Seoul, Republic of Korea; 6 Division of Life Sciences, College of Life Sciences and Biotechnology, Korea University, Seoul, Republic of Korea

**Keywords:** Autophagosome, ER stress, Migrasome, Retraction fiber, Cell death, ITGA5, TSPAN4

## Abstract

**Background:**

Glioblastoma (GBM) is more difficult to treat than other intractable adult tumors. The main reason that GBM is so difficult to treat is that it is highly infiltrative. Migrasomes are newly discovered membrane structures observed in migrating cells. Thus, they can be generated from GBM cells that have the ability to migrate along the brain parenchyma. However, the function of migrasomes has not yet been elucidated in GBM cells.

**Results:**

Here, we describe the composition and function of migrasomes generated along with GBM cell migration. Proteomic analysis revealed that LC3B-positive autophagosomes were abundant in the migrasomes of GBM cells. An increased number of migrasomes was observed following treatment with chloroquine (CQ) or inhibition of the expression of *STX17* and *SNAP29*, which are involved in autophagosome/lysosome fusion. Furthermore, depletion of *ITGA5* or *TSPAN4* did not relieve endoplasmic reticulum (ER) stress in cells, resulting in cell death.

**Conclusions:**

Taken together, our study suggests that increasing the number of autophagosomes, through inhibition of autophagosome/lysosome fusion, generates migrasomes that have the capacity to alleviate cellular stress.

**Supplementary Information:**

The online version contains supplementary material available at 10.1186/s12915-024-01829-w.

## Background

Glioblastoma (GBM) is a refractory tumor with an incidence of approximately ~ 16% among all adult primary brain tumors [1]. The median survival is only 12–14 months, and the only standard treatment is a combination of temozolomide and ionizing radiation therapy [2–4]. Recurrence of GBM, with a probability of up to 90%, also contributes to poor prognosis for patients with GBM [5]. Therefore, there have been many studies and efforts to treat GBM [6–8].

In 2015, the Li Yu group discovered a multivesicular body-like structure on the trailing edge of a migrating cell [9]. They named it the “migrasomes.” Migrasomes are generated during cell movement and are randomly distributed inside thin cellular structures called retraction fibers (RFs). These structures have been found in various types of cells, including normal rat kidney cells, and, recently, it has been suggested that their cell membranes are composed of tetraspanin- and cholesterol-enriched microdomains (TEMAs) [9, 10]. The cell membrane composed of TEMAs is guided by the stiffening phenomenon of the membrane caused by adhesion to the substrate on the bottom surface of the cultured cells, and this physical interaction creates a unique cell membrane structure [10]. This newly discovered cell membrane structure, which was thought to be reproduced only under artificial experimental conditions, reached a milestone when its formation and function were suggested in the zebrafish gastrulation phase [11]. That is, the migrasome supplying CXCL12 chemokine is generated in mesodermal and endodermal cells, and it was shown that chemotaxis by CXCL12 induces normal migration of dorsal forerunner cells (DFCs). As such, it has been established that migrasomes play an important role in the development of organisms, and the investigation of their function in vivo will enhance interest regarding their role in other fields of research. Recently, it has been demonstrated in neutrophils that mild mitochondrial stress leads to the incorporation of damaged mitochondria into the migrasomes, in a process called “mitocytosis” [12]. Simultaneously, interest in RF, which was only considered a pathway to construct the migrasome, is being highlighted [13].

During nutrient starvation or proteostatic stress, to survive under harsh conditions, cells restrict their energy use and produce essential components for maintaining cell viability using the autophagic pathway [14, 15]. During autophagy, cells produce double-membrane structures, namely phagophores, usually from the endoplasmic reticulum (ER), and multiple autophagy-related (ATG) genes are involved in this process [16]. It is known that about 20 ATG proteins are involved in the canonical autophagy pathway [17]. The core ATG proteins include the ULK1/2 kinase complex, ATG9A-associated vesicle trafficking system, and the PI3KC3 (Vps34 in yeast) complex [18]. These protein complexes play an essential role in phagophore nucleation during autophagy initiation. LC3 (*MAP1LC3A*, *MAP1LC3B*, and *MAP1LC3C*) is involved in the phagophore maturation stage [17, 18]. In yeast, it is an ubiquitin-like protein known as Atg8 [19]. The E1-like enzyme ATG7 adenylates ATG12 and is involved in the formation of ATG5-ATG12 conjugates, whose formation is mediated by the E2-like enzyme ATG10 [17, 20]. In addition, ATG7 plays a role in the phosphatidylethanolamine conjugation of LC3 and recruit LC3 to the phagophore, which is important for phagophore elongation [21]. Upon formation, autophagosomes fuse with the lysosome with the assistance of the SNARE protein complex called STX17-SNAP29-VAMP8, resulting in lysosomal degradation of substances within autophagosomes [22, 23].

Autophagy pathway acts as a stress-relief mechanism in cancer cells exposed to various types of stressors [24]. Cancer cells are exposed to environmental conditions such as chronic nutrient deficiency, hypoxia, and low pH due to abnormal blood vessel formation [25, 26]. In these environments, autophagy activation may play a crucial role in cancer progression and development. Autophagy is also known to be activated even after GBM treatment [27, 28]. Several studies have reported that the autophagy pathway suppresses GBM cell death [29, 30]. Treatment options for patients with GBM, such as arsenic trioxide (As_2_O_3_), temozolomide, rapamycin, and tamoxifen, promote autophagy to alleviate GBM cell death [31–34].

The functions of RF and the migrasome (R&M) have not been completely elucidated, and in particular, their role in cancer biology has not been investigated. In this study, we investigated the relationship between autophagosome and R&M formation and identified that R&M have a stress-relief function in brain tumor cells upon ER stress condition.

## Results

### Identification of cellular organelles enriched in GBM cell-derived R&Ms

**Fig. 1:**
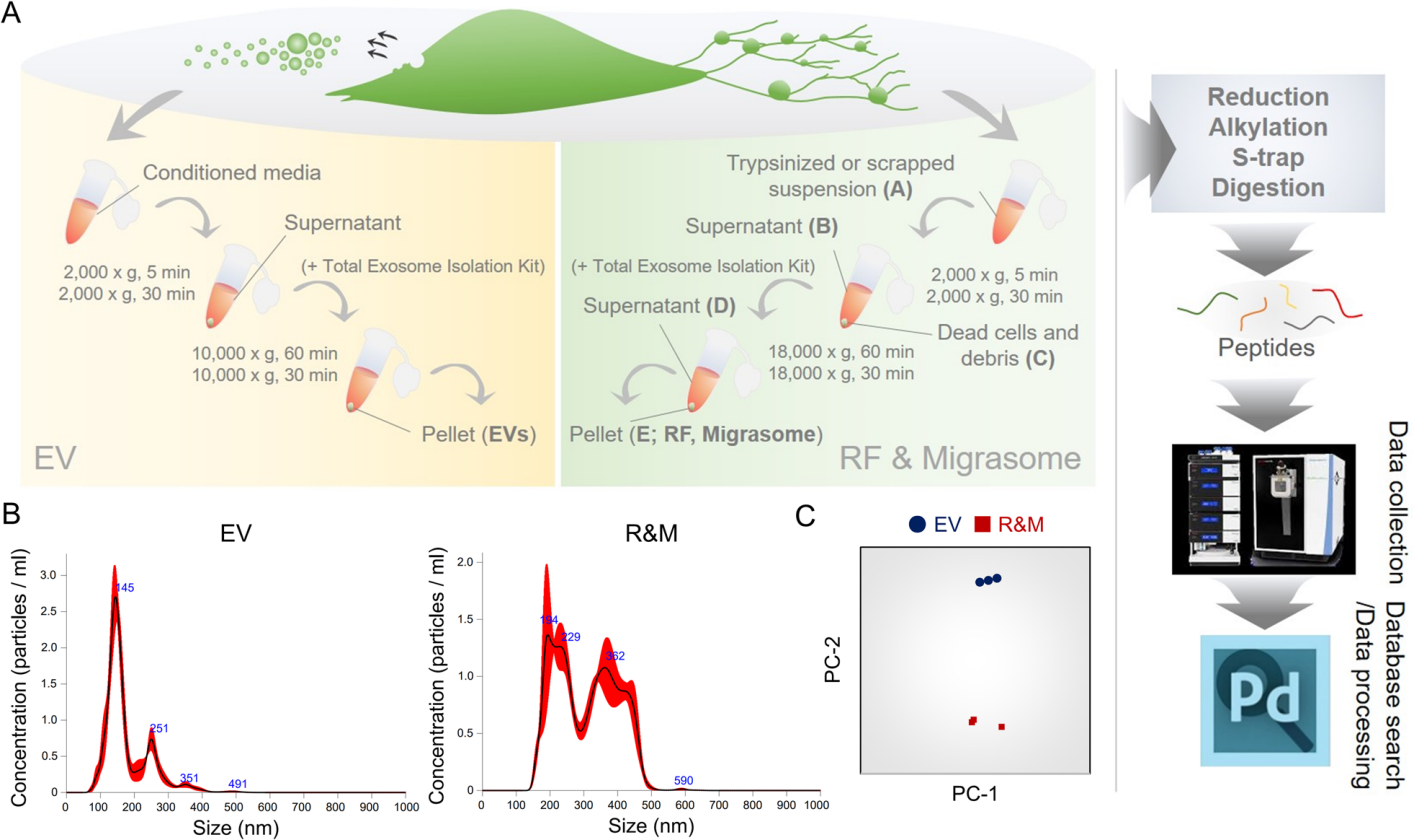
Identifying the presence of autophagosomes within retraction fiber & migrasome (R&M) of glioblastoma cells. **A** Purification procedures of tumor-derived extracellular vesicle (EV) and R&M. Both samples are purified and further analyzed using liquid chromatography-high-resolution mass spectrometry (LC-HRMS, Orbitrap Exploris 480, triple technical replicates). Samples were divided into three-technically replicated subsamples. For database searching and processing, we searched mapped protein sequences against SwissProt database (release v2019_06) and Proteome discoverer v2.2. **B** Nanoparticle tracking analysis used for quantifying and qualifying both EV and R&M. Three biological replicates were used for analysis. **C** Principal component analysis (PCA) shows that EVs and R&Ms have proteins with a distinctly different composition. PCA was performed using relative protein abundance values. The raw values of each protein abundance were converted to log2 values. Then, the amount of protein was corrected using the width adjustment method

**Fig. 2:**
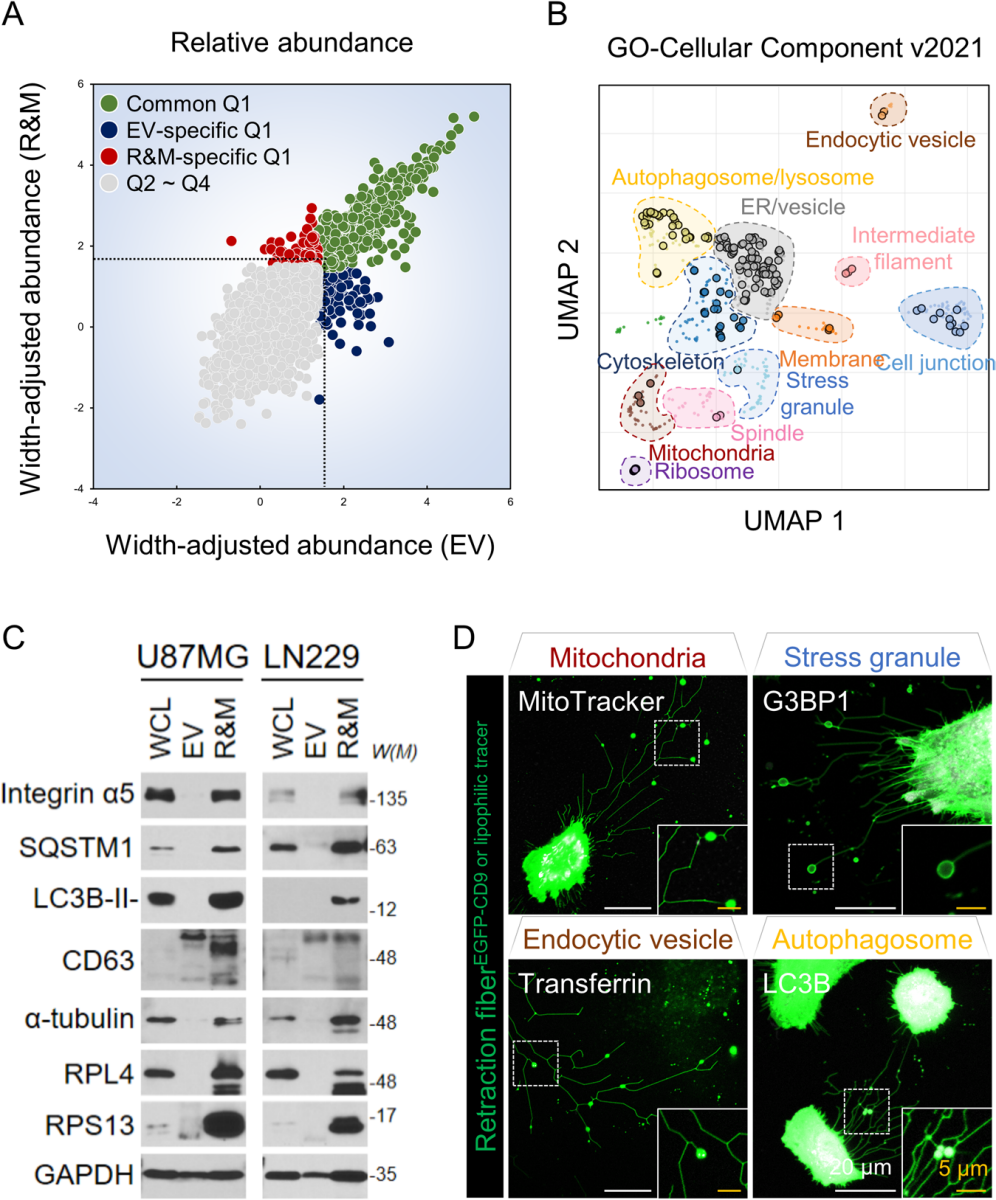
LC3B-positive autophagosomes are present within the retraction fiber and migrasome (R&M) of glioblastoma cells. **A** Based on the width-adjusted relative protein abundance values, the protein present in the first quartile or higher in each sample was specified as “Q1.” The remaining proteins, belonging to “Q2–Q4,” were excluded from the predominantly present proteins in each sample. **B** A scatter plot visualizing the result of Gene Ontology enrichment analysis (GO-Cellular Component v2021) for common Q1 and R&M-specific Q1 combined gene set. Plot was obtained from Enrichment Analysis Visualization Appyter v0.2.5, and it organized similar Gene Ontology gene sets into clusters using first two UMAP dimensions. We manually designated each cluster by follows: mitochondria, ribosome, stress granule, membrane, cell junction, intermediate filament, endocytic vesicle, ER/vesicle, cytoskeleton, spindle, and autophagosome/lysosome. **C** Western blotting of integrin α5, SQSTM1, LC3B, CD63, α-tubulin, RPL4, RPS13, and GAPDH proteins of whole cell lysate (WCL), EV, and R&M in U87MG and LN229 cells. *W(M)*, molecular weight. **D** Live-cell imaging for visualizing marker proteins of major cellular organelles derived from the result of (**B**). White, each marker of cellular organelles; MitoTracker Deep Red for visualizing mitochondria, tdTomato-G3BP1 for visualizing stress granules, EGFP-LC3B for visualizing autophagosomes, and transferrin 488 conjugate for visualizing endocytic vesicles. Green, EGFP-CD9 or DiO/DiI lipophilic tracer. Scale bars (white), 20 μm. Scale bars (yellow), 5 μm

RFs and migrasomes of GBM cells have been described in our previous study and by the Li Yu group [9, 35]. We anticipated that vigorously migrating GBM cells would generate migrasomes behind their trailing edge. We visualized RFs previously through CD9, a tetraspanin protein [10]. CD9 overexpression did not increase the number of migrasomes as in case of *TSPAN4* overexpression. We found that some cells were capable of generating migrasomes, even though other cells exhibited fewer migrasomes and only produced RFs (Additional file 1: Fig. S1A). In 2021, Wang et al. investigated the presence of damaged mitochondria within neutrophil migrasomes, both in vitro and in vivo [12]. Thus, we were intrigued as to which cellular organelles are abundantly located in migrasomes. For performing proteomic analysis, we purified crude migrasomes including RFs (i.e., R&M) along with extracellular vesicles (EVs) using serial centrifugation (Fig. 1A and Additional file 1: Fig. S1B–D). We then analyzed the cellular materials to distinguish them according to their size. The diameter of R&Ms were about 1.7-fold larger than that of EVs (EV: 177.7 ± 4.1 nm, R&M: 307.7 ± 6.0 nm), suggesting that crude migrasomes are large enough to distinguish them from EVs (Fig. 1B). Moreover, we further analyzed the relative abundance of proteins using principal component analysis based on liquid chromatography-high resolution mass spectrometry (LC-HRMS) (Fig. 1C). Technical replicates of each sample revealed a separate distribution in the analysis (Fig. 1C). We then examined the specific proteins enriched in each sample by calculating the relative abundance of proteins within the samples after adjusting the raw abundance value of the proteins. We defined the proteins enriched in each sample as those with a relative abundance value above the first quartile, “Q1” (Fig. 2A). By using combined gene sets from “common Q1” and “R&M-specific Q1,” we performed enrichment analysis using Enrichr, which is powered by Appyter [36, 37]. Consequently, enrichment analysis using GO-Cellular Component v2021 revealed enriched cellular organelles of combined gene sets to dimensionality-reduced uniform manifold approximation and projection (UMAP) (Fig. 2B). Among the diverse cellular organelles, autophagosome/lysosome, ER/vesicle, and cytoskeleton were the major organelles in the R&M samples.

Next, we investigated whether the R&M-enriched cellular organelles were truly located within R&M. Thus, we confirmed the expression of representative marker proteins using Western blot analysis and live-cell fluorescence imaging (Fig. 2C, D). Integrin α5, α-tubulin, CD63, and GAPDH are known marker proteins of migrasomes and exosomes [10, 35, 38]. Moreover, G3BP1, a major protein for nucleating stress granules [39], and mitochondria, which are known to be transported through retraction fibers in an anterograde manner during mitochondrial damage [12], were not observed in steady-state of cells. Transferrin, located in endocytic vesicles [40], has been detected in a few migrasomes. Interestingly, both LC3B-II, the lipidated form of LC3B (*MAP1LC3B* for gene name), and autophagosome bridging protein, SQSTM1, were highly expressed in R&M samples from both U87MG and LN229 cells. EGFP-LC3B was also strongly observed in live-cell imaging. Purified R&M samples were also prepared for detection using transmission electron microscopy. Some polysome-like structures, and lipid droplets, and autophagosome-like structures, were observed (Additional file 1: Fig. S1E). Together, we analyzed enriched cellular organelles within the R&M of GBM cells and observed high expression of LC3B and SQSTM1 proteins in R&M samples and intact migrasomes.

### Inhibition of autophagosome/lysosome fusion promotes the formation of R&Ms of GBM cell

**Fig. 3:**
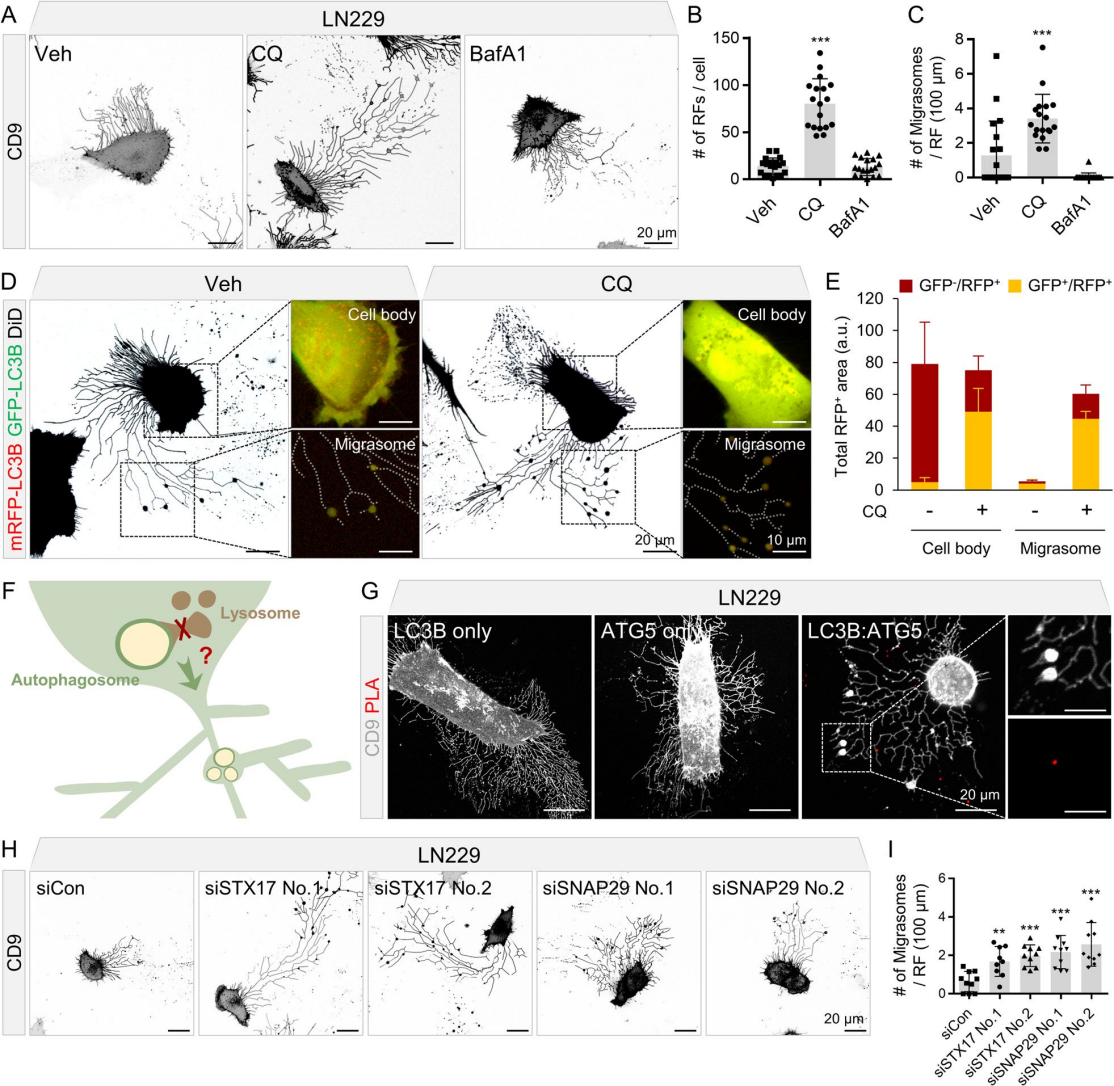
Inhibition of autophagosome/lysosome fusion induces retraction fiber and migrasome (R&M) formation. **A** Live-cell imaging for observing R&Ms formed in LN229 cells. Cells were treated with chloroquine (CQ; 50 μM, 12 h) or bafilomycin A1 (BafA1; 50 nM, 12 h). Gray, EGFP-CD9. Scale bars, 20 μm. **B** Quantification of the number of retraction fibers (RFs) per a cell. Image analyses were performed using results from (**A**). *n* = 19 for vehicle-treated condition, *n* = 18 for CQ- or BafA1-treated condition. **C** Quantification of the number of migrasomes per RF (100 μm). Image analyses were performed using the results from (**A)**. **D** Tandem-fluorescent LC3B was expressed in LN229 cell. Fluorescent signal observed in both cell body and migrasome was quantified respectively. Cells were treated with CQ. Red/green, mRFP-EGFP-LC3B. Black, DiD lipophilic tracer. Scale bars (black), 20 μm. Scale bars (white), 10 μm. **E** Quantification of **D**. Total RFP^+^ vesicle area was quantified in each condition. **F** Hypothetic graphical scheme of the relationship between autophagosome/lysosome fusion and R&M formation. **G** In situ proximity ligation assay (PLA) was performed to examine the interaction between LC3B and ATG5 in migrasomes of LN229. Cells were treated with CQ. Red, PLA signal. Gray, EGFP-CD9. Scale bars, 20 μm. Scale bars in cropped panels, 10 μm. **H** Live-cell imaging for observing R&M formed in LN229 cells expressing EGFP-CD9 after transfection of *STX17* or *SNAP29* siRNAs. Gray, EGFP-CD9. Scale bars, 20 μm. **I** Quantification of the number of migrasomes per RF (100 μm). Image analyses were performed using the results from (**H**). *n* = 10 for each image. The unpaired nonparametric Mann–Whitney *U*-test was used to analyze the statistical significance between each group. Data are expressed as mean ± SEM. In all data, **indicates *p* < 0.01, and ***indicates *p* < 0.001. The figure is representative of three-biological replicates with similar results

Next, we investigated whether the LC3B-II proteins found in the R&M samples of GBM cells were derived from the cellular autophagy process. As the phosphatidylethanolamine-conjugated LC3B-II proteins are accumulated in the circumstance of autophagic flux inhibition [41], we examined the formation of R&M in the presence of chloroquine (CQ) or bafilomycin A1 (BafA1), which disrupt autophagic degradation of cargos within autolysosomes under normal culture conditions. Both CQ- and BafA1-treated samples exhibited increased levels of LC3B-II (Additional file 1: Fig. S2A), as previously reported [41]. However, only CQ-treated LN229 cells generated more R&Ms compared to vehicle-treated cells (Fig. 3A–C). BafA1 is a vacuolar H^+^-ATPase inhibitor that inhibits the entry of H^+^ into lysosomes, thereby disrupting their function. CQ inhibits autophagosome/lysosome fusion; thus, pH-neutral LC3B-positive autophagosomes are concentrated in the native endomembrane system [41]. Therefore, we expressed a tandem-fluorescent LC3B construct [42] in LN229 cells to determine whether complete autophagosomes, which do not fuse with lysosomes, are observed within the R&M. Consequently, we observed increased number of LC3B-positive vesicles in both the cell body and migrasomes in the CQ-treated group (Fig. 3D, E). These LC3B-positive vesicles were also dual fluorescent positive, indicating pH-neutral autophagosomes existed within migrasomes. Next, we hypothesized that autophagosomes at the stage of phagophore maturation were observed within migrasomes (Fig. 3F). During phagophore maturation, the E1-like enzyme ATG7 mediates the conjugation of ATG5 and ATG12 to render phosphatidylethanolamine to LC3B-I proteins via complex formation with ATG16L [18, 20]. Thus, we performed a proximity ligation assay for LC3B and ATG5 (Fig. 3G). LC3B and ATG5 interact with each other; thus, autophagosomes, but not autolysosomes, are identified as sequestrated cellular vesicles during CQ treatment.

It is known that CQ abrogates autophagosome/lysosome fusion in association with the separation of STX17-positive vesicles from LAMP-2-positive lysosomes, which means that complete autophagosomes cannot undergo vesicular fusion with lysosomes in the presence of CQ [41]. Autophagosome/lysosome fusion is mediated by the STX17-SNAP29-VAMP8 complex [23]. STX17 is a SNARE protein that binds to the complete autophagosome and recruits SNAP29, which mediate complex formation of SNARE proteins with both autophagosomes (STX17) and lysosomes (VAMP8) [22, 23]. Thus, we depleted *STX17* and *SNAP29* expression in LN229 cells using small interfering RNA (siRNA) to investigate whether the inhibition of autophagosome/lysosome fusion by ablation of SNARE proteins induced the formation of R&M. As a result, the R&Ms of LN229 cells were increased in both *STX17* and *SNAP29* ablated condition (Fig. 3H, I and Additional file 1: Fig. S2B–D). Altogether, we identified that increased R&M after treatment with CQ is associated with inhibition of the autophagosome/lysosome fusion process, suggesting that autophagosomes that cannot be fuse with lysosomes are present within migrasomes.

### Endoplasmic reticulum (ER)-associated proteins are abundantly present within R&Ms in the context of ER stress

**Fig. 4:**
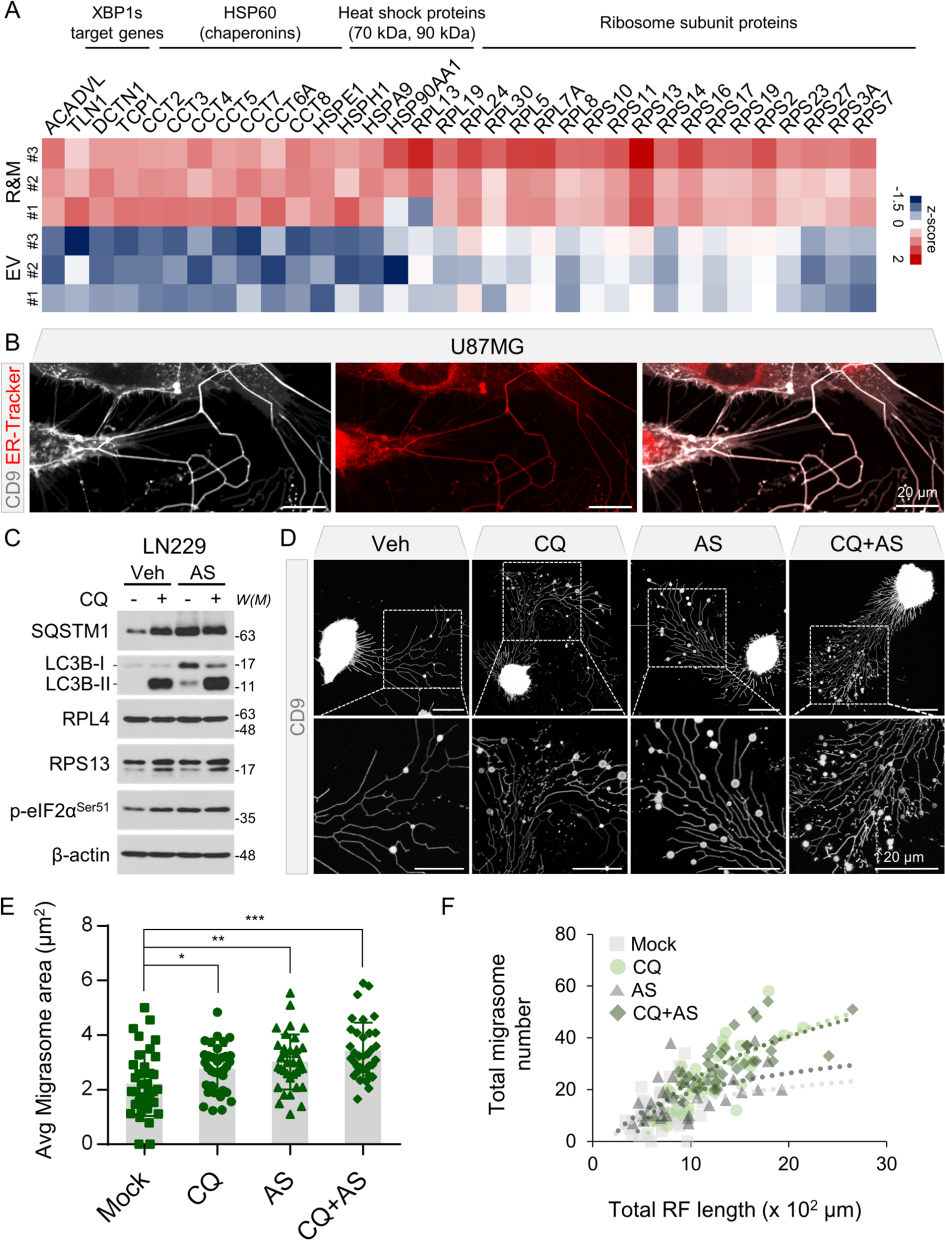
Endoplasmic reticulum (ER)-associated proteins are abundant cargo proteins present in retraction fiber & migrasome (R&M). **A** Heatmap represents highly enriched ER-associated proteins in R&M portion. Relative abundance values of both extracellular vesicle (EV) and R&M samples were standardized to z-score. **B** Live-cell imaging of U87MG cells for visualizing ER by using ER-Tracker Red. White, EGFP-CD9. Red, ER-Tracker. Scale bars, 20 μm. **C** Western blotting of SQSTM1, LC3B, RPL4, RPS13, p-eIF2α (Ser51), and β-actin proteins after treatment of chloroquine (CQ; 50 μM, 12 h) or NaAsO_2_ (AS; 10 μM, 12 h) in LN229 cells. *W(M)*, molecular weight. **D** Live-cell imaging for quantifying R&M formation in CQ (50 μM, 12 h) or AS (10 μM, 12 h) treatment condition. Green, EGFP-CD9. White, tdTomato-RPS13. Scale bars, 20 μm. **E** Quantification of average migrasome area (μm^2^) in data from (**D**). LN229 cell was used for observation and image analyses. *Indicates *p* < 0.05; **indicates *p* < 0.01; ***indicates *p* < 0.001. Data are expressed as mean ± SEM. The unpaired nonparametric Mann–Whitney *U*-test was used to analyze the statistical significance between each group (*n* = 35). **F** Quantification of total migrasome number and total RF length (× 10^2^ μm) in data from (**D**)

Tumor cells require vigorous amounts of building blocks to survive under stressful conditions, such as nutrient depletion and hypoxic conditions [14, 15]. Thus, these cells activate the autophagy pathway to enhance cell viability. Several macromolecules and organelles are involved in autophagic degradation [16–18]. We investigated cargo proteins that were abundantly present in R&M samples. We found that ER-associated proteins, including HSP60 families (i.e., chaperonins), heat shock 70- or 90-kDa proteins, and ribosomal subunit proteins, were specifically present in the R&M samples (Fig. 4A). Moreover, we observed that R&Ms produced from GBM cells were positive for ER-Tracker (Fig. 4B). These results suggest that R&Ms are abundant with ER-associated proteins, which have function in protein folding. Meanwhile, in the context of proteotoxic stress, such as low-dose arsenic stress, transient ER stress occurs concomitantly with the inhibition of autophagy [43]. Thus, we hypothesized that ER-associated R&Ms can be further increased by proteostatic stress, such as arsenic stress. We treated LN229 cells expressing EGFP-CD9 and tdTomato-RPS13 with CQ, sodium arsenite (NaAsO_2_; AS), and a combination of both. Both CQ and AS upregulated p-eIF2a levels, indicating that ER stress was induced, and CQ and AS treatment had the same inhibitory effects on autophagy flux; that is, both conditions inhibited the autophagy pathway and prevented autophagosomes from fusing with lysosomes (Fig. 4C). It has been reported that arsenic-induced autophagy inhibition is mediated by increased O-GlcNAcylation of the SNAP29 protein [44]. Thus, in this case, AS may inhibit autophagosome/lysosome fusion via the same mechanism as CQ. As we observed that cells in the AS treatment condition exhibited an increased number and size of migrasomes, including the number of RFs (Fig. 4D–F). We also detected dual-fluorescent LC3B-positive autophagosomes within migrasomes (Additional file 1: Fig. S2E–F).

Next, we hypothesized that we could force cells to produce more R&M by reinforcing autophagic flux and inhibiting autophagy. Thus, we induced autophagy by treating cells with Torin-1, an mTORC1/2 inhibitor. Interestingly, treatment with Torin-1 by itself was unable to promote R&M formation (Additional file 1: Fig. S3A–D). Instead, RF formation was reduced, which is presumed to be due to enhanced autophagy flux degrading ER/Golgi substrates, potentially leading to reduced RF formation. However, in the case of co-treatment with Torin-1 and CQ, we observed an increase in the amount of R&Ms compared to the single treatment condition, indicating that disrupted autophagic flux under enhanced autophagy may further induce R&M formation (Additional file 1: Fig. S3A–D).

Altogether, we determined that ribosomal subunit proteins were predominantly enriched within the R&Ms of GBM cells. Proteotoxic stress, such as that caused by AS, induces ER stress with an inhibitory effect on autophagy, suggesting that ribosomes are degraded under stressful conditions However, these proteins cannot be completely degraded and left in autophagosomes (i.e., located within migrasomes) upon exposure to CQ or AS due to their inhibitory effect on autophagy pathway.

### Inhibition of R&M formation induces cell death through out of control of ER stress

**Fig. 5:**
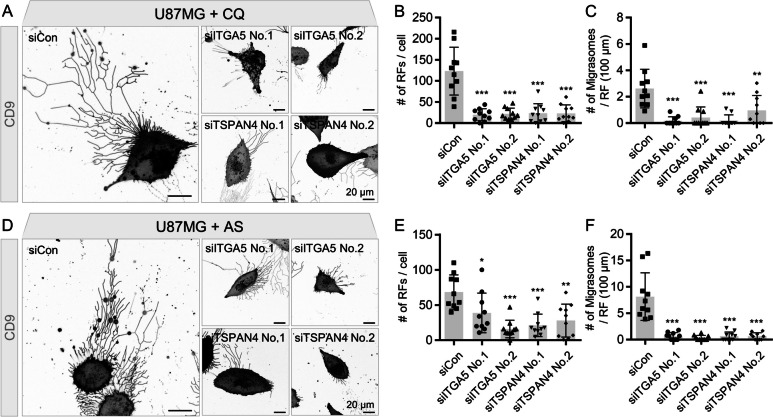
Genetic ablation of ITGA5 or TSPAN4 decrease retraction fiber & migrasome (R&M) formation. **A** Live-cell imaging of *ITGA5* or *TSPAN4* siRNA-transfected U87MG cells. Cells were treated with chloroquine (CQ; 50 μM, 12 h) or NaAsO_2_ (AS; 10 μM, 12 h). Scale bars, 20 μm. **B** Quantification of the number of RFs per cell. Image analyses were performed using results from (**A**). ***Indicates *p* < 0.001. Data are expressed as mean ± SEM. The unpaired nonparametric Mann–Whitney *U*-test was used to analyze the statistical significance between each group (*n* = 10). **C** Quantification of the number of migrasomes per RF (100 μm). Image analyses were performed using results from (**A**). **Indicates *p* < 0.01. ***Indicates *p* < 0.001. Data are expressed as mean ± SEM. The unpaired nonparametric Mann–Whitney *U*-test was used to analyze the statistical significance between each group (*n* = 10). **D** Live-cell imaging of *ITGA5* or *TSPAN4* siRNA-transfected U87MG cells. Cells were treated with NaAsO_2_ (AS; 10 μM, 12 h). Scale bars, 20 μm. **E** Quantification of the number of RFs per a cell. Image analyses were performed using results from (**D**). *Indicates *p* < 0.05. **indicates *p* < 0.01. ***Indicates *p* < 0.001. Data are expressed as mean ± SEM. The unpaired nonparametric Mann–Whitney *U*-test was used to analyze the statistical significance between each group (*n* = 10). **F** Quantification of the number of migrasomes per RF (100 μm). Image analyses were performed using results from (**D**). ***Indicates *p* < 0.001. Data are expressed as mean ± SEM. The unpaired nonparametric Mann–Whitney *U*-test was used to analyze the statistical significance between each group (*n* = 10)

**Fig. 6:**
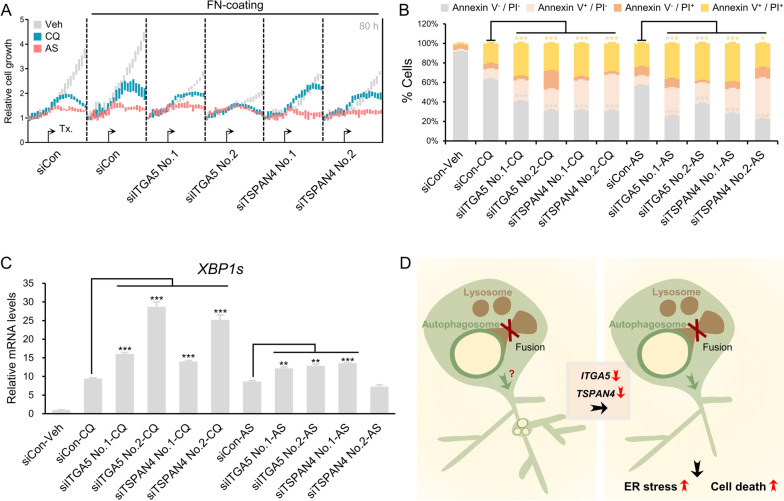
Cells restrained retraction fiber & migrasome (R&M) formation cannot alleviate ER stress. **A** Cell growth rate of *ITGA5*- or *TSPAN4*-depleted U87MG cells in CQ (50 μM) or AS (10 μM)-treated conditions. Relative cell growth was calculated based on cell confluence mask analyzed by using IncuCyte ZOOM. **B** Annexin V/PI staining for analyzing cell death of *ITGA5*- or *TSPAN4*-depleted U87MG cells in CQ- or AS-treated conditions. Cells were harvested after 72-h treatment of CQ or AS. *Indicates *p* < 0.05; ***indicates *p* < 0.001. Data are expressed as mean ± SEM. Student’s *t*-test was used to analyze the statistical significance between each population (*n* = 3 for technical replicates). **C** qRT-PCR data for evaluating mRNA levels of spliced *XBP1*. *Indicates *p* < 0.05. **Indicates *p* < 0.01. ***Indicates *p* < 0.001. Data are expressed as mean ± SEM. Student’s *t*-test was used to analyze the statistical significance between each group (*n* = 3). **D** Graphical summary of study. In *ITGA5*- or *TSPAN4*-depleted condition, cells have a decreased number of R&Ms and lower capability to relieve ER stress, resulting in increased apoptosis

Previously, it has been reported that the autophagy pathway is associated with ER stress [45]. Moreover, inhibition of autophagosome/lysosome fusion induced ER stress concomitant with R&M formation (Fig. 4C). Next, we disturbed R&M formation by decreasing the levels of *ITGA5* and *TSPAN4* [10, 35, 38] and regulators of R&M formation to investigate the function of R&M in ER stress condition. By depleting the levels of both *ITGA5* and *TSPAN4* using siRNA, we observed a lower number of R&M in U87MG cells than in control cells (Fig. 5A-F and Additional file 1: Fig. S3E). Furthermore, we examined whether reduced R&M formation renders cells vulnerable to ER stress. Thus, we analyzed cellular phenotypes after stimulation with CQ or AS to induce ER stress concomitant with the inhibition of autophagosome/lysosome fusion. Cells with depleted levels *ITGA5* or *TSPAN4* exhibited lower growth rates and more dead cells after exposure to CQ or AS than control cells (Fig. 6A, B). As cell death is not directly associated with the knockdown of *ITGA5* or *TSPAN4*, we hypothesized that these genes are involved in ER stress-induced cell death via R&M formation. Thus, we identified the cellular states when cells were exposed to stress stimuli, in this case CQ or AS treatment. We confirmed ER stress markers in CQ- or AS-treated *ITGA5* or *TSPAN4* knockdown cells. We found that cells could not alleviate ER stress in the case of *ITGA5* or *TSPAN4* knockdown, exhibiting increased levels of spliced *XBP1* mRNA (Fig. 6C). Taken together, we revealed that decreased R&M formation did not relieve ER stress in GBM cells exposed to CQ or AS (Fig. 6D).

## Discussion

In vivo environments always contain a matrix to which cells can attach [46, 47]. Therefore, in such an environment, the formation of R&M occurs actively. In particular, tumor cells have a high potential to form cell membrane structures through cell migration or invasion. Our study revealed that the R&M formed by GBM cells was generated on the trailing side of the migrating cells and contained autophagosomes.

According to previous reports, there are several papers reporting that BafA1 not only inhibits autolysosome function through V-ATPase inhibition but also inhibits autophagosome/lysosome fusion [48, 49]. However, this part remains controversial, at least in mammalian cells; it is more convincing that it does not have the effect of inhibiting the fusion of STX17-positive mature autophagosome based on latest report [41]. We tried to solve that controversial part by adding *STX17* and *SNAP29* knockdown experiments after the experiment using CQ, and, consequently, it was observed that knockdown of *STX17* and *SNAP29* inhibited autophagosome/lysosome fusion and increased migrasome production in GBM cells.

In terms of migrasome production, we suggested the possibility of association between autophagosome and migrasome formation through some experiments. However, there is a limitation in that the relationship between autophagy and migrasome is incompletely demonstrated. We believe that complementary experiments with autophagy induction and autophagy-related genes are important that can reveal whether autophagy and migrasome formation are related. This should be clearly explained in future study.

RFs appears to be a residue of actin filaments that the migrating cells have not retrieved. Actin filaments are known to interact with various proteins, and it was found that Arp2, one of the proteins constituting the branch of actin filaments, interacts with Atg9, a major factor involved in phagophore elongation [50]. The RF region, where the migrasome is present, is a junction point and seems to play a similar role to an actin filament branch. It is not known why these branch points are generated, but if it is assumed that the cytoskeleton is left behind by a migrating cell, the branch point will be a part of the Arp2/3 complex. The formation and maturation of autophagosomes cannot be considered in isolation, because of their association with the cytoskeleton [51]. It has been reported that LC3 helps the actin nucleation function of JMY by binding to the LC3-interacting region (LIR) of the JMY protein during starvation [52]. Additionally, during the autophagy initiation phase, WHAMM, known as nucleation-promoting factor (NRF), recruits the Arp2/3 complex and is involved in autophagosome formation through the assembly of actin filaments aside from the ER [53]. That is, the interaction between autophagosomes and the actin cytoskeleton is an essential process in the formation of autophagosomes, and the formation of migrasomes, which seem to have autophagosomes inherent in the RF passage, which is an actin-based structure, is a natural process.

In contrast, autophagosomes can fuse with vesicles constituting other endomembrane systems. A representative example is the fusion with endosome, which is called amphisome. The amphisome is LC3 positive and harbors endosomal markers [54]. The autophagosome bound to the multivesicular body shows a propensity to co-localize with exosomal markers. This type of fusion occurs in a unique environment such as shear stress [55]. Amphisomes have macromolecules such as DNA and histones, separate from other EVs, and can be considered the origin of extracellular dsDNA [54]. Whether the LC3-positive vesicle found in the migrasome is an autophagosome or a different type of LC3-positive vesicle structure requires more precise identification.

## Conclusions

Vigorously migrating GBM cells have a potential to generate R&Ms. Migrasomes are formed by inhibition of autophagosome/lysosome fusion, which is important for degrading cellular cargos in stress condition. Furthermore, we confirmed that ER-associated proteins were abundantly present within R&Ms. Moreover, the increased ER stress can generate much more R&Ms in GBM cells. Genetic ablation of *ITGA5* or *TSPAN4* decreased the R&M formation. We demonstrated that decreased R&Ms promote unfolded protein response, thus enhancing cell death in stressful conditions.

The formation of autophagosomes and autophagy pathway is important mechanisms for alleviating the accumulation of stress damage in cancer cells [56, 57]. In addition, instead of processing the accumulated autophagosome using the energy of the cell itself, the method of perishing from the cell body through the migrasome generated from cell movement is considered to support the survival of cancer cells.

Obviously, cells that make up any tissue do not exist statically. In this system, cells show dynamic movement, and in this process, more or less traces will be left, depending on the physical factors of the surrounding environment. Perhaps the cells were in constant communication through their dynamic movements and the substances they left behind, RF and migrasome. In the tumor environment, this membranous material exchange creates a favorable environment for cancer cells.

## Methods

### Cell lines and cell culture

Human GBM cell lines U87MG (human origin, male, GBM, RRID: CVCL_0022) and LN229 (human origin, female, GBM, RRID: CVCL_0393) were purchased from the American-Type Culture Collection (ATCC, Manassas, VA, USA). All cell lines were authenticated using short tandem repeat (STR) profiling and tested for mycoplasma contamination. The cells were cultured in high-glucose Dulbecco’s Modified Eagle Medium (DMEM; Lonza, Basel, SWZ) supplemented with 10% fetal bovine serum (FBS; HyClone, Logan, USA), 1% penicillin/streptomycin (P/S; HyClone), and 2 mM L-glutamine (HyClone) at 37 °C with 5% CO_2_ and 95% humidity.

### Proximity ligation assay (PLA)

PLA was performed to detect LC3B-ATG5 binding in GBM cells. U87MG and LN229 cells (3 × 10^4^) expressing EGFP-CD9 were seeded on fibronectin (FN, 10 μg/ml; Sigma-Aldrich, Cat. No. F0895, St. Louis, MO, USA)-coated coverslips in 48-well plates for 1 day. The cells were then fixed in 4% PFA for 15 min at RT. Cells were washed twice with 1 × PBS. The fixed cells were permeabilized using 0.05% saponin (Sigma-Aldrich, Cat. No. S7900) in PBS for 30 min. Samples were stained using primary antibodies for 3 h at RT followed by Duo-Link in situ PLA probes with anti-rabbit MINUS (Sigma-Aldrich, Cat. No. DUO92005), anti-mouse PLUS (Sigma-Aldrich, Cat. No. DUO92001), and Duo-Link in situ detection reagents Red (Sigma-Aldrich, Cat. No. DUO92008). Anti-LC3B antibody (rabbit polyclonal antibody, 1:100; Novus Biologicals, Cat. No. NB100-2220, Centennial, CO, USA, RRID:AB_10003146) and anti-ATG5 antibody (mouse monoclonal antibody, 1:100; Santa Cruz Biotechnology, Cat. No. sc-133158, Dallas, TX, USA, RRID:AB_2243288) were used as primary antibodies. Mounted samples were observed using confocal laser scanning microscopy (CLSM) (LSM700; Carl Zeiss, Jena, DEU, Plan-Apochromat × 63/1.40 Oil DIC M27).

### Plasmids and lentivirus infection

EGFP-CD9 were cloned into the pCDH-CMV-MCS-EF1a-Puro vector for overexpression. mRFP-EGFP-LC3B was cloned into the pLL-CMV-Puro vector for overexpression. For transient expression, the plasmids were transfected using LipoJet (SignaGen Laboratories, Frederick, MD, USA). To construct a stable cell line, we performed lentivirus infection. To produce the lentivirus, each expression vector was transfected into HEK293T cells with second-generation lentiviral packaging plasmids pdR8.91 and pVSV-G using LipoJet. Twenty-four hours after transfection, the culture medium was harvested, incubated with Lenti-X concentrator (Clontech Laboratories, Cat. No. h631231, Mountain View, CA, USA), and centrifuged to obtain the concentrated lentivirus. The cells were infected with the lentiviruses in the presence of 6 μg/mL polybrene (Sigma-Aldrich, Cat. No. H9268) for 24 h.

### siRNA transfection

To perform siRNA-mediated knockdown of genes, we transfected siRNAs using ScreenFectA Transfection Reagent (Wako Pure Chemical Industries, Cat. No. 293–73201, Osaka, Japan) according to the manufacturer’s instructions. All siRNAs were synthesized from BIONEER (Double-Strand RNA Oligo; BIONEER Inc., Daejeon, Korea).

The siRNA information is as follows: human *STX17*, 5′-GAACACAAGUAUAUCAAGA-3′ and 5′-CUGGAAAUGUGAAAACUGA-3′; human *SNAP29*, 5′-GCAAAAUGCUUAUUAGAGU-3′ and 5′-GAUUUCCACUCUAUUGUGA-3′; human *ITGA5*, 5′-CUCCUAUAUGUGACCAGAG-3′ and 5′-GUUUCACAGUGGAACUUCA-3′; and human *TSPAN4*, 5′-CCAUCGCCAUCCUCUUCUU-3′ and 5′-GUGGACCCCUCACCUACAU-3′.

### Electron microscopy imaging

Scanning electron microscopy was performed as previously described [58]. Briefly, purified U87MG R&Ms were fixed using 2.5% glutaraldehyde (Sigma-Aldrich, Cat. No. 354400) in 0.1-M phosphate buffer (pH 7.4) overnight at 4 °C. U87MG-derived brain tumor-bearing mice were anesthetized and perfused by the same fixative buffer. The samples were post-fixed with 2% osmium tetroxide for 2 h. After standard dehydration in ethanol series (60%, 70%, 80%, 90%, 95%, 100%, and 100%, each for 20 min, and then 100% for 30 min), the samples were immersed in t-butyl alcohol for 20 min twice. Next, the samples were dried using a freeze dryer (Hitachi ES-2030; Hitachi, Tokyo, Japan) and coated with platinum in ion sputter (Hitachi E-1045; Hitachi). The cells were observed using a scanning electron microscope (Hitachi S-4700; Hitachi).

For transmission electron microscopy, U87MG and LN229 cells were cultured on coverslips coated by FN. Next day, the experiment was performed as previously described [59]. Briefly, the cells were fixed by 2% paraformaldehyde (PFA)/2.5% glutaraldehyde in 0.1-M phosphate buffer (pH 7.4) overnight at 4 °C. The samples were post-fixed with 2% osmium tetroxide for 2 h. After standard dehydration in ethanol series (60%, 70%, 80%, 90%, 95%, 100%, and 100% each for 20 min and 100% for 30 min), the dehydrated samples were suspended in propylene oxide for 20 min. Epon infiltration was performed by the serial incubation of 2:1, 1:1, and 1:2 of propylene oxide:epon mixture for 1 h each. Next, the samples were embedded in epoxy resin mixture and polymerized at 60 °C in a dry oven for 48 h. Polymerized blocks were fine trimmed into the paramedian lobule. Semi-thin Sects. (1 μm) were obtained using an ultramicrotome (Leica UC7; Leica, Wetzlar, DEU) and observed via toluidine blue staining. Sections were mounted on formvar-coated one-hole grids and subsequently post-stained with heavy metals. Serial images were randomly obtained from the samples using a transmission electron microscope (Hitachi H-7650; Hitachi).

### Live-cell fluorescence imaging and quantification of R&Ms

GBM cells (3 × 10^4^) were seeded on FN-coated coverslips in 24-well plates or 35-mm confocal dishes for 1 day. The cells were then imaged using live-cell confocal microscopy. Live cell images were obtained using CLSM (LSM700; Carl Zeiss, Plan-Apochromat × 63/1.40 Oil DIC M27, × 10–35 images of U87MG cells, and × 10–35 images of LN229 cells were obtained by 1 × magnification at × 63, ZEN acquisition software version 2018, blue edition) with live cell incubator (Chamlide TC-FC5N; Live Cell Instrument (LCI), Seoul, Korea). The images were randomly taken for each sample. We used ImageJ software v.1.52a (National Institute of Health (NIH), Bethesda, MD, USA) to calculate the RFs, their junctions, total length, and the number of migrasomes of all images by using “Ridge Detection” plugin [60]. CQ (Selleckchem, Cat. No. NSC-187208, Houston, TX, USA), AS (Alfa Aesar, Cat. No. 041533.AP, 0.1N standardized solution, Ward Hill, MA, USA), and BafA1 (Sigma-Aldrich, Cat. No. B1793) were used for experiments.

### Purification of EV and R&M

EVs were purified using Total Exosome Isolation Reagent (Thermo Fisher Scientific, Cat. No. 4478359) according to manufacturer’s instruction. Also, crude migrasome extraction was performed based on Li Yu group’s method [9]. In brief, U87MG and LN229 cells seeded on FN-coated 100-mm culture dishes were cultured in DMEM supplemented with 10% EV-free FBS. The EV-free FBS was obtained from FBS (HyClone) by ultracentrifuge at 100,000 × g for 18 h at 4 °C, followed by filtration of supernatant FBS through a syringe filter (0.1-μm pore size; Satorius, Cat. No. 16553-K, Goettingen, DEU). The medium was collected and centrifuged at 2000 rpm for 4 min 4 °C to remove cell bodies and then 4000 rpm for 20 min 4 °C to remove cell debris for obtaining EVs. Remaining cells on culture dishes were detached by 0.015% Trypsin-EDTA or scrapping and centrifuged at 2000 rpm for 4 min 4 °C to remove cell bodies and then 4000 rpm for 20 min 4 °C to remove cell debris for obtaining R&Ms. The supernatant was mixed in a ratio 2:1 with Total Exosome Isolation Reagent. The mixture was incubated for overnight at 4 °C. Next day, the mixture was centrifuged at 10,000 × g or 18,000 × g for 1 h at 4 °C to collect EV or R&M, respectively. The pellet was washed by PBS twice and then resuspended in 100–200 μL of PBS and prepared for nanoparticle tracking analysis, electron microscope sampling, and Western blot analysis.

### Nanoparticle tracking analysis (NTA)

NTA measurement was performed using NanoSight NS300 (Malvern Panalytical, Malvern, GBR) as previously described [61]. All EV and R&M samples were diluted in PBS filtered through a syringe filter (0.1-μm pore size; Satorius) to a final volume of 1 mL. Ideal measurement concentrations were found by multiple testing the ideal particle per frame value (20–50 particles/frame). Following settings were set according to the manufacturer’s software manual (NanoSight NS300 User Manual, MAN0541-01-EN-00, 2017): camera level was increased until all particles were clearly visible not exceeding a particle signal saturation over 20% (EV: level 13; R&M: level 9). The ideal detection threshold was determined to include as many particles as possible with the restrictions that 10–100 red crosses were counted, while only < 10% were not associated with distinct particles. Blue cross count was limited to 5. Autofocus was adapted so that unclear particles were omitted. For each measurement, four time captures of 60-s videos were obtained under the following conditions: temperature: 25 °C and Syringe speed: 40 μL/s. After capture, the videos have been analyzed by the in-build NanoSight software NTA 3.4 Build 3.4.003 with a detection threshold of 7. Hardware is as follows: embedded laser: Red; camera is as follows: sCMOS. The number of completed tracks in NTA measurements was always greater than the proposed minimum of 1000 in order to minimize data distortion based on single large particles.

### Sample preparation for proteomics

EV and R&M samples were resuspended in 400 μL of 5% SDS in 50-mM TEAB (pH 7.55), and dithiothreitol was added to a final concentration of 20 mM for 10 min at 95 °C to reduce disulfide bonds. Reduced samples were then incubated with 40-mM iodoacetamide for 30 min at room temperature in the dark. By a tenfold dilution of 12% phosphoric acid, acidified samples were loaded onto S-Trap macro (EV) or mini (R&M) columns (ProtiFi, Farmingdale, NY, USA; Cat. No.: CO2-macro-80 or CO2-mini-80). We treated suspension-trapping (S-trap) proteolysis according to the manufacturer’s protocol, followed by the addition of 1:20 Lys-C/trypsin mixture and incubation for 16 h at 37 °C [62]. The eluted peptide mixture was lyophilized using a cold trap and stored at − 80 °C until use.

### Nano-LC–ESI–MS/MS analysis

The LC system was an Dionex UltiMate 3000 RSLCnano system (Thermo Fisher Scientific). Mobile phase A was 0.1% formic acid, and 5% DMSO in water and mobile phase B was 0.1% formic acid, 5% DMSO, and 80% acetonitrile in water. Samples were reconstituted with 25 μL of mobile phase A, injected with a full sample loop injection of 5 μL into a C18 PepMap trap column (20 × 100 μm i.d., 5 μm, 100 Å; Thermo Fisher Scientific), and separated in EASY-Spray column (500 × 75 μm i.d., 2 μm, 100 Å; Thermo Fisher Scientific) over 200 min (250 nL/min) at 50 °C. The column was priory equilibrated with 95% mobile phase A and 5% mobile phase B. A gradient of 5–40% B for 150 min, 40–95% for 2 min, 95% for 23 min, 95–5% B for 10 min, and 5% B for 15 min were applied. The LC system was coupled to an Orbitrap Exploris 480 mass spectrometer (Thermo Fisher Scientific) with a nano EASY-Spray™ source. For all experiments, spray voltage was set to 2.1 kV, ion transfer tube temperature to 290 °C, and a radio frequency funnel level to 40%. Full scans were made at a resolution of 60,000. MS1 AGC target was set to 3,000,000 charges, with a maximum injection time of 25 ms. Scan ranges used (in m/z) were as follows: 350–1600. Precursor fragmentation was achieved through higher-energy collision disassociation (HCD) with a normalized collision energy of 30 and an isolation width of 1.3 m/z. Full scans were made at a resolution of 15,000. MS2 AGC target was set to 2,000,000. Charges 2–5 were considered for MS/MS, with a dynamic exclusion of 20 ms. Loop counts were set to 12. MS2 resolution was set to 15,000. Maximum MS2 injection times were as follows: 22 ms.

### Protein identification by database search

Individual raw files acquired MS analysis and were retrieved against the reviewed Human Uniprot-SwissProt protein database (released on June 2019) [63] using the SEQUEST-HT on Proteome Discoverer (Version 2.2, Thermo Fisher Scientific). Search parameters used were as follows: 10-ppm tolerance for precursor ion mass and 0.02 Da for fragmentation mass. Trypsin peptides tolerate up to two false cleavages. Carbamidomethylation of cysteines was set as fixed modification, and N-terminal acetylation and methionine oxidation were set as variable modifications. The false discovery rate was calculated using the target-decoy search strategy, and the peptides within 1% of the FDR were selected using the post-processing semi-supervised learning tool Percolator [64] based on the SEQUEST result. Label-free quantitation of proteins was calculated using the precursor ion peak intensity for unique and razor peptides of each protein and excluded peptides with methionine oxidation.

### Western blotting

Western blot was performed to analyze the protein expression. Briefly, cell extracts were prepared using RIPA lysis buffer (150-mM sodium chloride, 1% NP-40, 0.1% SDS, 50-mM Tris, pH 7.4) containing 1-mM β-glycerophosphate, 2.5-mM sodium pyrophosphate, 1-mM sodium fluoride, 1-mM sodium orthovanadate, and protease inhibitor (Roche, Cat. No. 11836170001, Basel, Switzerland). The protein concentration was quantified using the Bio-Rad Protein Assay Dye Reagent Concentrate (Bio-Rad, Cat. No. 5000006, Hercules, CA, USA) according to the manufacturer’s instructions. Proteins were resolved by SDS-PAGE and then transferred to a immobilon-P polyvinylidene fluoride membrane (Merck Millipore, Cat. No. IPVH00010, Danvers, MA, USA). Membranes were blocked with 5% nonfat milk and incubated with the primary antibody. Membranes were then incubated with horseradish peroxidase-conjugated anti-IgG secondary antibody (Pierce Biotechnology, Rockford, IL, USA) and visualized using the SuperSignal West Pico PLUS Chemiluminescent Substrate (Thermo Fisher Scientific, Cat. No. 34580). Primary antibodies used for Western blot analysis are as follows: anti-SQSTM1 (p62; mouse monoclonal antibody, 1:1000; Santa Cruz Biotechnology, Cat. No. sc-28359, RRID:AB_628279), anti-β-actin (mouse monoclonal antibody, 1:10,000; Santa Cruz Biotechnology, Cat. No. sc-47778, RRID:AB_626632), anti-LC3B (rabbit polyclonal antibody, Novus Biologicals, Cat. No. NB100-2220, RRID:AB_10003146), anti-RPS13 (mouse monoclonal antibody, 1:500; Santa Cruz Biotechnology, Cat. No. sc-398690), anti-RPL4 (mouse monoclonal antibody, 1:500; Santa Cruz Biotechnology, Cat. No. sc-100838, RRID:AB_2181910), anti-α-tubulin (mouse monoclonal antibody, 1:10,000; Sigma-Aldrich, Cat. No. T6199, RRID:AB_477583), anti-integrin α5 (rabbit monoclonal antibody, 1:1000; Abcam, Cat. No. ab150361, RRID:AB_2631309), anti-CD63 (rabbit polyclonal antibody, 1:1000; Santa Cruz Biotechnology, Cat. No. sc-15363, RRID:AB_648179), anti-p-eIF2α (Ser51) (rabbit polyclonal antibody, 1:1000; Cell signaling, Cat. No. 9721, Danvers, MA, USA, RRID:AB_330951).

### Quantitative reverse transcription-PCR (qRT-PCR)

The qRT-PCR was performed to determine mRNA levels. Briefly, total RNA was isolated from cells using the QIAzol lysis reagent (QIAGEN, Cat. No. 79306, Valencia, CA, USA) according to the manufacturer’s instructions. The 1 U of DNase I, RNase-free (Thermo Fisher Scientific, Cat. No. EN0525), was added to 1 μg of template RNA and incubated for 30 min at 37 °C. For inactivating DNase I, 50 mM of EDTA was treated and heated at 65 °C for 10 min. DNase I-treated RNA was utilized as a template for synthesizing complementary DNA (cDNA) using the RevertAid First-Strand cDNA Synthesis Kit (Thermo Fisher Scientific, Cat. No. K1622) according to the manufacturer’s instructions. The qRT-PCR analysis was performed using the Takara Bio SYBR Premix Ex Taq (Takara, Cat. No. RR420A, JPN) and CFX096 (Bio-Rad). The expression levels of each target gene were normalized to that of 18S rRNA. The primers used for the analyses are listed in Additional file 1: Table S1.

### Cell death assay

Cell death was evaluated using flow cytometry analysis. Briefly, U87MG (2.5 × 10^5^) cells were seeded in a FN-coated 60-mm culture dish and incubated with CQ or AS for 72 h. Cell pellets were centrifuged at 3500 rpm for 5 min and then stained with APC Annexin V (BD Biosciences Pharmingen, Cat. No. 550474, Franklin Lakes, NJ, USA). Each cell suspension (100 μL in binding buffer) stained with 10-μL APC Annexin V and 20-μL propidium iodide (PI; 50 μg/mL) was gently mixed and incubated for 15 min. Next, an additional 200 μL of binding buffer was added, and then the cells were immediately analyzed using a FACSVerse apparatus. Non-apoptotic cell death (cells stained with PI alone), early apoptosis (cells positive for APC annexin V alone), and late apoptosis (cells positive for PI and APC annexin V) were observed.

### Statistical analyses

All data from the experiments, shown as bar graphs, are presented as mean ± SEM. Statistical analysis was performed by using the unpaired nonparametric Mann-Whitney *U*-test or two-tailed Student’s *t*-tests. Values of *p* < 0.05 or *p* < 0.01 were considered statistically significant for different experiments, as indicated in the figure legends.

### Supplementary Information

**Additional file 1: Fig. S1.** Retraction fiber & migrasome (R&M) of glioblastoma cells and quality control of purified R&Ms. **Fig. S2.** Inhibition of autophagosome/lysosome fusion by chemical drugs and genetic ablation. **Fig. S3.** Reinforcement of autophagy under the disturbed autophagic flux induces R&M formation. **Fig. S4.** Uncropped blots for Western blot experiments. **Table S1.** The primers used in this study.

**Additional file 2.** Raw data points for n < 6 data values. A few more additional raw data points are also provided.

## Data Availability

All data generated or analyzed during this study are included in this published article, its supplementary information files, and publicly available repositories. We deposited raw MS data from the instrument and spectral libraries with ProteomeXchange [65]. Consortium (http://proteomecentral.proteomexchange.org) through the Proteomics Identification Database (PRIDE) [66] and allied repository with identifier PXD047816. Uncropped Western blots are provided in Additional file 1: Fig. S4. Raw data values for some figures which have less than 6 samples are provided in Additional file 2. Additional file 2 also includes raw data values for Fig.  2A, Fig.  3 A–C, and Fig. S2E.

## Abbreviations

AS: Sodium arsenite
ATG: Autophagy related
BafA1: Bafilomycin A1
CQ: Chloroquine
CRISPR: Clustered regularly interspaced short palindromic repeat
DFC: Dorsal forerunner cell
dsDNA: Double-stranded DNA
ER: Endoplasmic reticulum
FN: Fibronectin
GBM: Glioblastoma
GO: Gene Ontology
LC-HRMS: Liquid chromatography-high resolution mass spectrometry
LIR: LC3-interacting region
NOS: Nitric oxide synthase
NRF: Nucleation-promoting factor
NTA: Nanoparticle tracking analysis
PI: Propidium iodide
PLA: Proximity ligation assay
RF: Retraction fiber
R&M: Retraction fiber & migrasome
sgRNA: Single-guide RNA
siRNA: Small interfering RNA
TEMA: Tetraspanin- and cholesterol-enriched microdomain
WGA: Wheat germ agglutinin
