# Supplementary material for: Migrasomal autophagosomes relieve endoplasmic reticulum stress in glioblastoma cells
Source: BMC Biol. 2024 Jan 30;22:23. doi: 10.1186/s12915-024-01829-w (PMC10826056; doi:10.1186/s12915-024-01829-w)
Supplement: Supplementary file 1 — Additional file 1: Fig. S1. Retraction fiber & migrasome (R&M) of glioblastoma cells and quality control of purified R&Ms. Fig. S2. Inhibition of autophagosome/lysosome fusion by chemical drugs and genetic ablation. Fig. S3. Reinforcement of autophagy under the disturbed autophagic flux induces R&M formation. Fig. S4. Uncropped blots for Western blot experiments. Table S1. The primers used in this study. [file 12915_2024_1829_MOESM1_ESM.pdf]

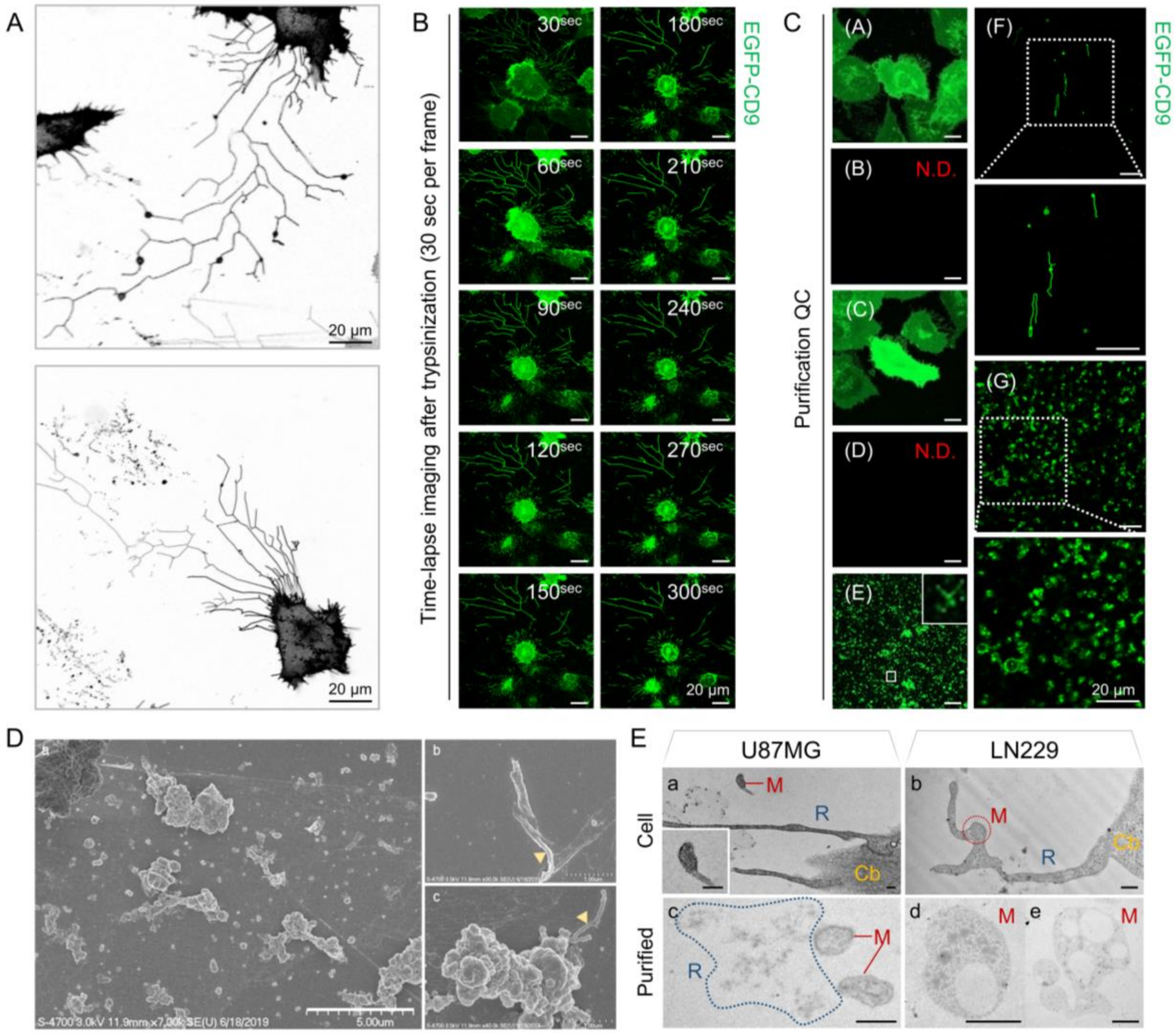

Fig. S1. Retraction fiber & migrasome (R&M) of glioblastoma cells and quality control of purified R&Ms. **A** LN229 cell expressing EGFP-CD9 produced R&Ms (upper panel) and produced few migrasomes (lower panel). Gray, EGFP-CD9. Scale bar, 20 µm. **B** Time-lapsed live-cell imaging (captured in every 30 sec) of U87MG EGFP-CD9 cells after treatment of 0.015% trypsin/EDTA. Green, EGFP-CD9. Scale bars, 20 µm. **C** Quality control of the R&M purification stages in Fig. 1A. Each quality control sample were obtained and observed immediately. The samples designated in each image (“A” ~ “E”) are paralleled samples described in R&M purification stages of Fig. 1A. The image “F” represents the debris obtained after trypsinization procedure and the image “G” represents the purified R&M obtained by using Exosome Purification Reagent. Green, EGFP-CD9. Scale bars, 20 µm. **D** Scanning

electron microscope images of purified R&M. From “a” to “f”, irregular and atypical purified R&M samples are shown. Yellow arrows represent the retraction fiber-like structures. Scale bars are indicated in each figure. **E** Transmission electron microscope images of attached cells and purified R&M. From “a” to “e”, transmission electron microscope imaging of attached cells and purified U87MG-R&M and LN229-R&M were presented. R, retraction fiber; M, migrasome; Cb, Cell body. Scale bars, 200 μm.

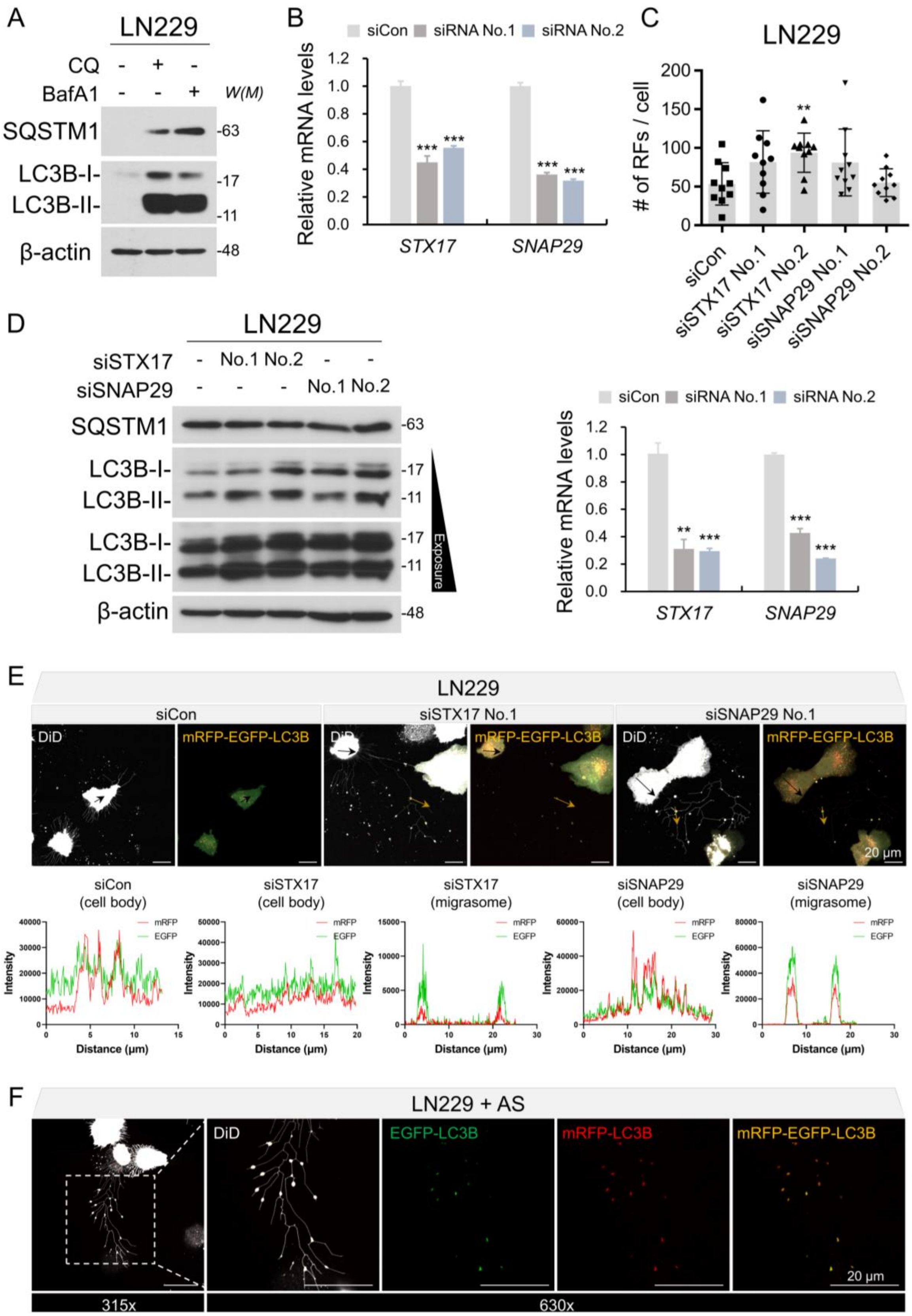
A
LN229
CQ - + -
BafA1 - - +
W(M)
SQSTM1 -63
LC3B-I- -17
LC3B-II- -11
β-actin -48
B
siCon siRNA No.1 siRNA No.2
Relative mRNA levels
STX17
SNAP29
C
LN229
# of RFs / cell
siCon
siSTX17 No.1
siSTX17 No.2
siSNAP29 No.1
siSNAP29 No.2
D
LN229
siSTX17 - No.1 No.2 - -
siSNAP29 - - - No.1 No.2
SQSTM1 -63
LC3B-I- -17
LC3B-II- -11
Exposure
β-actin -48
siCon siRNA No.1 siRNA No.2
Relative mRNA levels
STX17
SNAP29
E
LN229
siCon
siSTX17 No.1
siSNAP29 No.1
DiD
mRFP-EGFP-LC3B
20 μm
siCon (cell body)
siSTX17 (cell body)
siSTX17 (migrasome)
siSNAP29 (cell body)
siSNAP29 (migrasome)
mRFP
EGFP
Intensity
Distance (μm)
F
LN229 + AS
DiD
EGFP-LC3B
mRFP-LC3B
mRFP-EGFP-LC3B
20 μm
315x
630x

Fig. S2. Inhibition of autophagosome/lysosome fusion by chemical drugs and genetic ablation. **A** Western blotting of SQSTM1, LC3B, and β-actin proteins in and LN229 cells. Cells were treated with chloroquine (CQ; 50 μM, 12 h) or bafilomycin A1 (BafA1; 50 nM, 12 h). *W(M),* molecular weight. **B** qRT-PCR experiment for quantify mRNA levels of *STX17* and *SNAP29* in LN229 cells transfected with *STX17* or *SNAP29* siRNAs. *** indicates $p < 0.001$. Data are expressed as mean ± SEM. Student's t-test was used to analyze the statistical significance between each group (n = 3). **C** Quantification of the number of RFs per a cell. Image analyses were performed using results from Fig. 3H. ** indicates $p < 0.01$. Data are expressed as mean ± SEM. The unpaired nonparametric Mann-Whitney U test was used to analyze the statistical significance between each group (n = 10). **D** Western blotting of SQSTM1, LC3B, and β-actin proteins in and LN229 cells with siSTX17 or siSNAP29 (left panel). qRT-PCR experiment set of cells used in Western blotting for quantify mRNA levels of *STX17* and *SNAP29*. ** indicates $p < 0.01$. *** indicates $p < 0.001$ (right panel). Data are expressed as mean ± SEM. Student's t-test was used to analyze the statistical significance between each group (n = 3). **E** Tandem-fluorescent LC3B expressed in LN229 cells were used for performing knockdown of *STX17* and *SNAP29*. GFP^-/RFP^+ vesicles represent autolysosomes and GFP^+/RFP^+ vesicles represent autophagosomes (upper panel). Scale bars, 20 μm. Colocalization analysis was performed for investigating colocalized pattern of GFP and RFP (lower panel). **F** Tandem-fluorescent LC3B was expressed in LN229 cell. Live-cell imaging performed in $NaAsO_2$ (AS; 10 μM, 12 h) treatment condition. Scale bars, 20 μm.

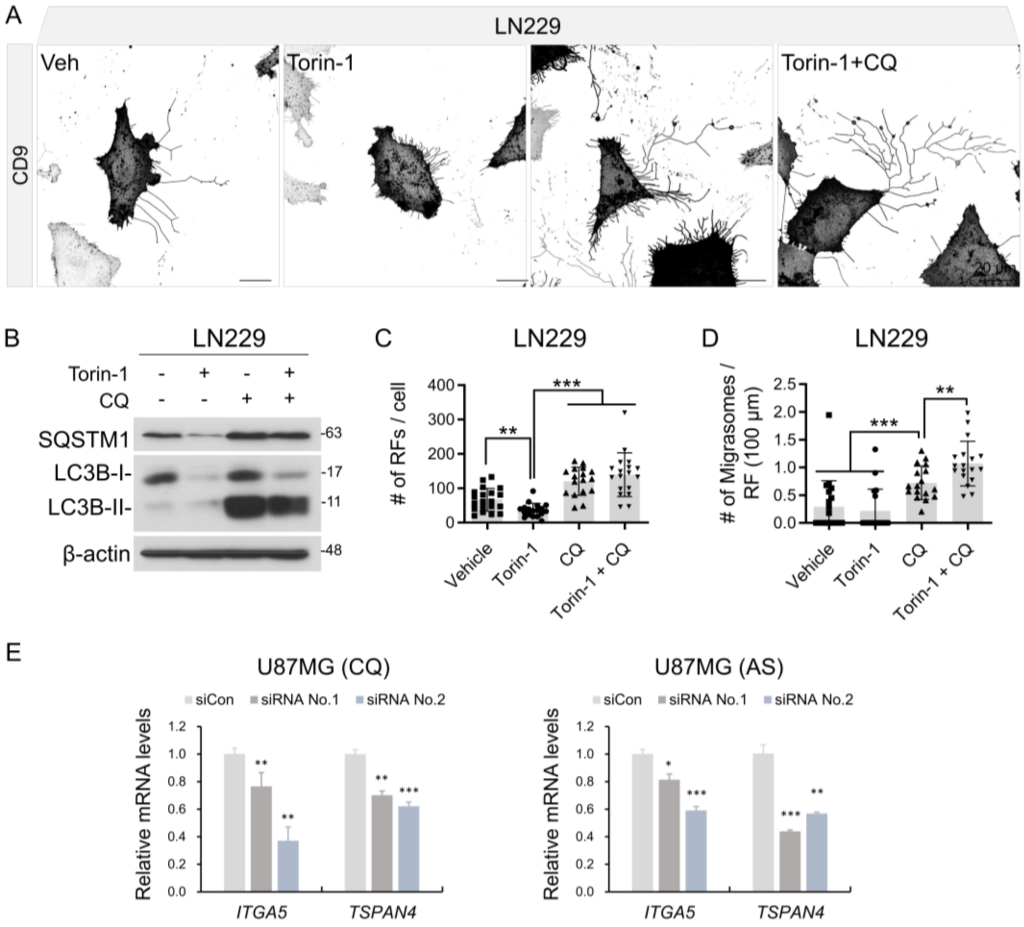

Fig. S3. Reinforcement of autophagy under the disturbed autophagic flux induces R&M formation. **A** Live-cell imaging of LN229 cells. Cells were treated with Torin-1 (250 nM, 2 h) and/or chloroquine (CQ; 50 µM, 12 h). The number of cells imaged for quantifying retraction fibers (RFs) and migrasomes are as follows: n = 20 for DMSO, n = 18 for Torin-1, n = 17 for CQ, and n = 18 for Torin-1/CQ. Scale bars, 20 µm. **B** Western blotting of SQSTM1, LC3B, and β-actin under treatment with Torin-1 and/or CQ. **C** Quantification of the number of RFs per a cell. Image analyses were performed using results from Fig. S3A. **D** Quantification of the number of migrasomes per RF (100 µm). Image analyses were performed using the results from Fig. S3A. **E** qRT-PCR experiment set of cells used in Fig. 5 and 6 for determining the knockdown efficiency of both *ITGA5* and *TSPAN4*. * indicates $p < 0.05$. ** indicates $p < 0.01$. *** indicates $p < 0.001$ (n = 3).

A Uncropped blots (Fig. 2C)

Integrin α5

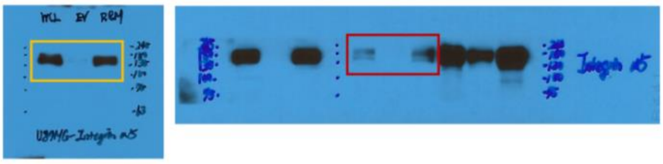

SQSTM1 (p62)

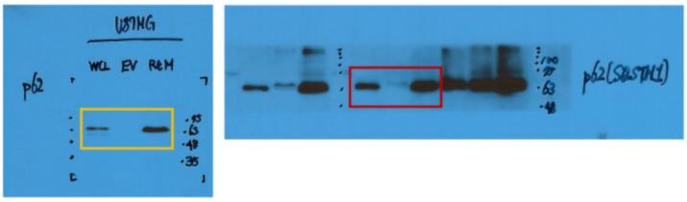

LC3B

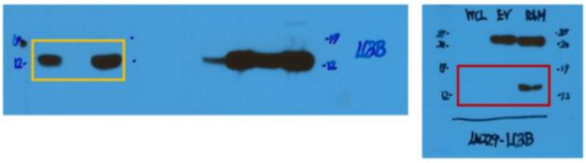

CD63

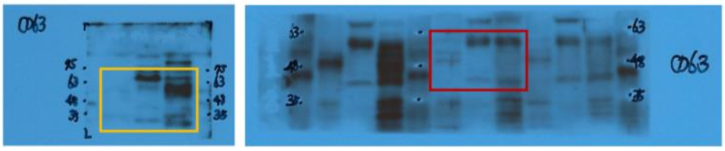

α-tubulin

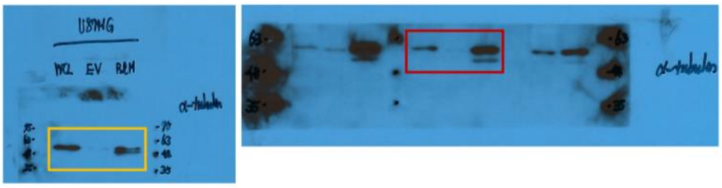

RPL4

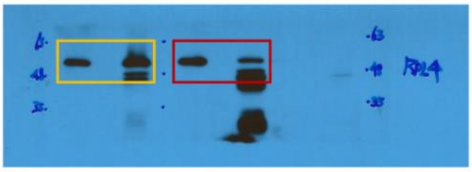

RPS13

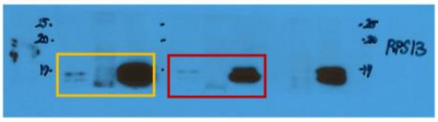

GAPDH

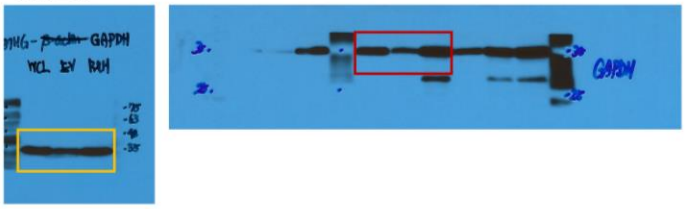

B Uncropped blots (Fig. 4C)

SQSTM1 (p62), RPL4

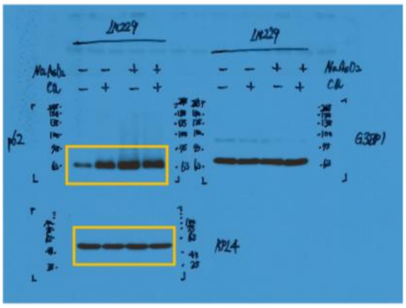

LC3B, β-actin

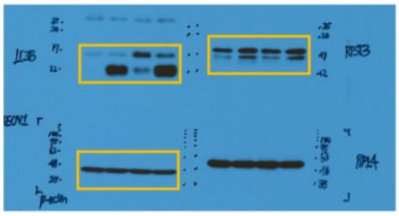

p-eIF2α

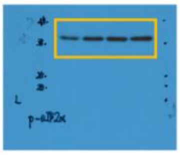

C Uncropped blots (Fig. S2A)

SQSTM1 (p62)

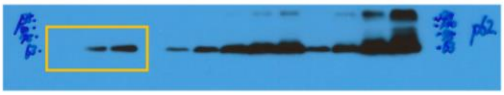

LC3B

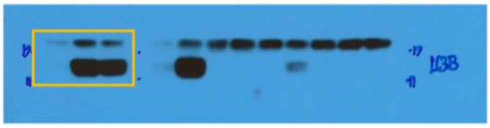

β-actin

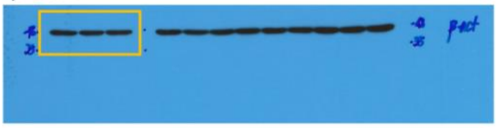

D Uncropped blots (Fig. S2D)

SQSTM1 (p62)

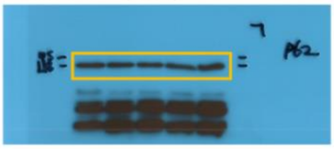

LC3B (short expose)

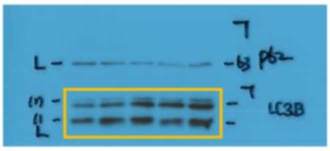

LC3B (long expose)

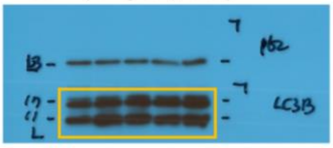

β-actin

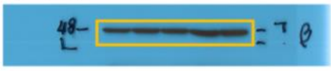

E Uncropped blots (Fig. S3B)

SQSTM1 (p62), β-actin

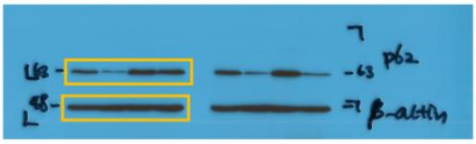

LC3B

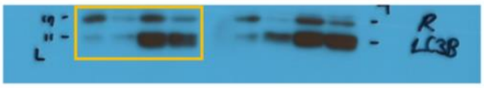

Fig. S4. Uncropped blots for Western blot experiments. **A** Uncropped blots of Fig. 2C. Western blotting was conducted by immunoblotting against the following antibodies: integrin α5, SQSTM1 (p62), LC3B, α-tubulin, RPL4, RPS13, and GAPDH. **B** Uncropped blots of Fig. 4C. Western blotting was performed by immunoblotting against the following antibodies: SQSTM1 (p62), LC3B, p-eIF2α (Ser51), and β-actin. **C** Uncropped blots of Fig. S2A. Western blotting was conducted by immunoblotting against the following antibodies: SQSTM1 (p62), LC3B, and β-actin. **D** Uncropped blot images of Fig. S2D. Western experiments was performed by using the following antibodies: SQSTM1 (p62), LC3B, and β-actin. **E** Uncropped blot images of Fig. S3B. Western blotting was performed by using the following antibodies: SQSTM1 (p62), LC3B, and β-actin.

Gene	Species	Direction	Sequence (5′-3′)
18S rRNA	*Homo Sapiens*	Forward	CAGCCACCCGAGATTGAGCA
		Reverse	TAGTAGCGACGGGCGGTGTG
ITGA5	*Homo Sapiens*	Forward	AGCAAGAGCCGGATAGAGGA
		Reverse	TCAGGGCATTCTTGTCACCC
TSPAN4	*Homo Sapiens*	Forward	TGGGTGCCATCAAGGAGAAC
		Reverse	CTTGTCCGTGTAGGCGAAGA
STX17	*Homo Sapiens*	Forward	GGGGAATGGTGTGGTGCTAA
		Reverse	TAGACATGCAGTTGGGCTGG
SNAP29	*Homo Sapiens*	Forward	CTGGCCCTCATGTACGAGTC
		Reverse	AGGGTGCCATTCTGTTCAGG
XBP1s	*Homo Sapiens*	Forward	GCTGAGTCCGCAGCAGGT
		Reverse	CTGGGTCCAAGTTGTCCAGAAT

Table S1. The primers used in this study.
